# Prostate cancer management: long-term beliefs, epidemic developments in the early twenty-first century and 3PM dimensional solutions

**DOI:** 10.1007/s13167-020-00214-1

**Published:** 2020-06-26

**Authors:** Radek Kucera, Ladislav Pecen, Ondrej Topolcan, Anshu Raj Dahal, Vincenzo Costigliola, Frank A. Giordano, Olga Golubnitschaja

**Affiliations:** 1 Department of Immunochemistry Diagnostics, University Hospital and Faculty of Medicine, Pilsen, Czech Republic; 2 Center of Molecular Biotechnology, Rheinische Friedrich-Wilhelms-Universität Bonn, Bonn, Germany; 3 European Medical Association, Brussels, Belgium; 4 Department of Radiation Oncology, University Hospital Bonn, Rheinische Friedrich-Wilhelms-Universität Bonn, Bonn, Germany; 5 Predictive, Preventive and Personalised (3P) Medicine, Department of Radiation Oncology, University Hospital Bonn, Rheinische Friedrich-Wilhelms-Universität Bonn, Sigmund-Freud-Str. 25, 53105 Bonn, Germany

**Keywords:** Predictive preventive personalised medicine (PPPM, 3PM), Prostate cancer (PCa), Multi-factorial systemic disease, Radical prostatectomy, Castration resistant, Comorbidities, Malignancy, Incidence, Mortality, Disease manifestation, Circulating tumour cells (CTC), Age, Elderly, Patient stratification, Aggressive metastatic disease, Multi-omics, Biomarker patterns, Survival, Liquid biopsy, Modifiable risk factors, Risk assessment, Multi-parametric analysis, Human development index, Adolescence, Young population, Family history, Genetic, Race, Ethnicity, Socio-economic factors, Urinary tract infection, Sexually transmitted diseases, Lifestyle, Diet, Obesity, Body mass index BMI, Oxidative stress, Inflammation, Physical activity, Smoking, Alcohol consumption, Sleep disorders, Saturated fat, Choline, Vasectomy, Insulin-like growth factor, Vitamins A, C, D, E, and K, Folate, Lycopene, Garlic, Green tea, Coffee, Curcumin, Stillbenes, Ellargic acid, Sulphorapane, Quercetin, Economy, Ethics, Life quality, Microcirculation, Systemic hypoxic condition, Ischemic lesions, Gut microbiota, Prebiotics, Probiotics, Fruits, Vegetables, Fish, Meat, Personalised nutrition, Toxic environment, Trace elements, Selenium/selenite, Apoptosis resistance, Androgen dependent, Prognosis, PSA screening, Urology, Prostate cancer antigen 3, miRNA, Aetiology, Indicator, Hybrid imaging, PET/MRI, Transrectal ultrasound, C-index, Lactate dehydrogenase, Bone-specific alkaline phosphatase, Roadmap, Individualised patient profile, Adapted treatment algorithms, Trends

## Abstract

In the early twenty-first century, societies around the world are facing the paradoxal epidemic development of PCa as a non-communicable disease. PCa is the most frequently diagnosed cancer for men in several countries such as the USA. Permanently improving diagnostics and treatments in the PCa management causes an impressive divergence between, on one hand, permanently increasing numbers of diagnosed PCa cases and, on the other hand, stable or even slightly decreasing mortality rates. Still, aspects listed below are waiting for innovate solutions in the context of predictive approaches, targeted prevention and personalisation of medical care (PPPM / 3PM).

A. PCa belongs to the cancer types with the highest incidence worldwide. Corresponding economic burden is enormous. Moreover, the costs of treating PCa are currently increasing more quickly than those of any other cancer. Implementing individualised patient profiles and adapted treatment algorithms would make currently too heterogeneous landscape of PCa treatment costs more transparent providing clear “road map” for the cost saving.
B. PCa is a systemic multi-factorial disease. Consequently, predictive diagnostics by liquid biopsy analysis is instrumental for the disease prediction, targeted prevention and curative treatments at early stages.
C. The incidence of metastasising PCa is rapidly increasing particularly in younger populations. Exemplified by trends observed in the USA, prognosis is that the annual burden will increase by over 40% in 2025. To this end, one of the evident deficits is the reactive character of medical services currently provided to populations. Innovative screening programmes might be useful to identify persons in suboptimal health conditions before the clinical onset of metastasising PCa. Strong predisposition to systemic hypoxic conditions and ischemic lesions (e.g. characteristic for individuals with Flammer syndrome phenotype) and low-grade inflammation might be indicative for specific phenotyping and genotyping in metastasising PCa screening and disease management. Predictive liquid biopsy tests for CTC enumeration and their molecular characterisation are considered to be useful for secondary prevention of metastatic disease in PCa patients.
D. Particular rapidly increasing PCa incidence rates are characteristic for adolescents and young adults aged 15–40 years. Patients with early onset prostate cancer pose unique challenges; multi-factorial risks for these trends are proposed. Consequently, multi-level diagnostics including phenotyping and multi-omics are considered to be the most appropriate tool for the risk assessment, prediction and prognosis. Accumulating evidence suggests that early onset prostate cancer is a distinct phenotype from both aetiological and clinical perspectives deserving particular attention from view point of 3P medical approaches.

## Introduction

The prostate cancer (PCa) management was for a long-time preoccupied by quite conservative beliefs which can be shortly summarised as follows: PCa is a life non-threatened disease of organ-specific aetiology (rather than systemic) being characteristic for elderly and, therefore, could be easily detected by PSA screening applied to ageing male population followed by radical prostatectomy as an optimal approach to treat PCa.

**Fig. 1:**
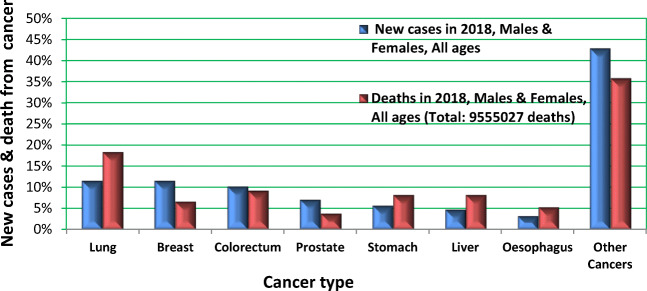
Cancer statistics monitored by GLOBOCAN in 2018 [1, 2]

Consequently, in the early twenty-first century, societies around the world are facing the paradoxal epidemic development of PCa as a non-communicable disease (Fig. 1) [1]. PCa is demonstrated amongst the most frequent cancers globally [2].

**Fig. 2:**
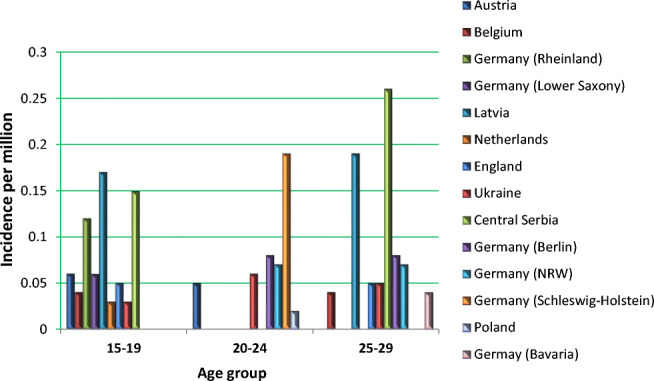
PCa in young European populations: in years 2004–2010 incidence rates per million of inhabitants [1, 14]

PCa is the second most common cause of cancer death in men after lung cancer [3] and the most frequently diagnosed cancer for men in several countries such as the USA [4]. In 2018, 1,276,106 new PCa cases were diagnosed and 358,989 related deaths were recorded worldwide [5]. PCa is a highly heterogeneous disease, ranging from a clinically insignificant to a highly aggressive castration-resistant type of tumours [6]. Further, three types of malignancies have been demonstrated to spread the highest amount of circulating tumour cells (CTC) in blood, namely, breast, prostate and lung [7]. To this end, CTC is a reliable indicator for developing metastatic disease in PCa, amongst other cancers [8–10]. The incidence of metastatic PCa is rapidly increasing in populations worldwide. Multi-factorial risks for metastatic disease in aggressive PCa subtypes have been proposed by several research groups [11]. Finally, PCa in young populations is considered an emerging challenge [12]. For instance, in the UK, a disproportional increase has been recorded between increasing incidence since the 1990s: for men aged 25–49 years by over 400%, compared with 285% for men aged 50–59 year, 142% for men aged 60–69 years, 42 and 23% for men aged 70–79 and 80+ years, respectively [13]. Incidence rates per million of inhabitants recorded in years 2004–2010 for young European populations are presented in Fig. 2 [14]. Multi-factorial risks underlying these trends have been proposed [1].

**Fig. 3:**
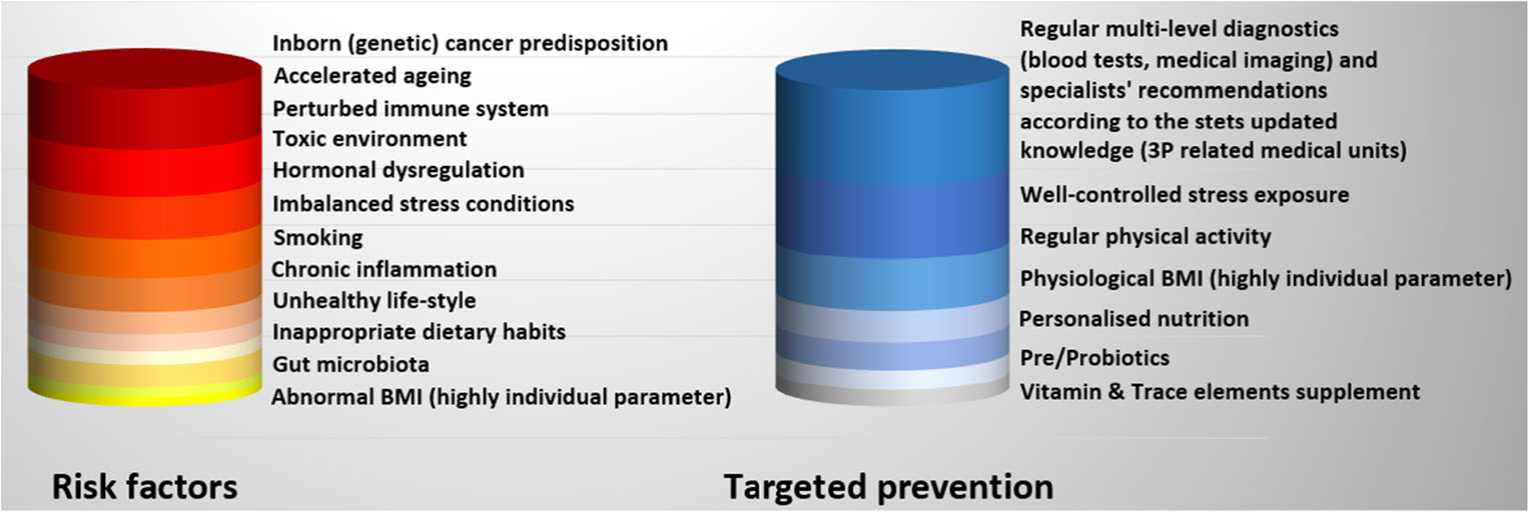
PCa-relevant risk factors and targeted prevention

Similarly to already recognised and currently extensively discussed epidemic developments of other non-communicable diseases such as type 2 diabetes [15] and breast cancer [16, 17], the PCa management paradigm obviously requires a fundamental revision meeting needs of young populations [18], individuals at risk by both genetic and modifiable factors [19, 20] as well as stratified patient cohorts benefiting from individualised treatment algorithms [21]. The article summarises knowledge accumulated in regard to the PCa-relevant non-modifiable and modifiable risk factors, highlights current deficits in PCa managements and presents innovative solutions in the context of predictive, preventive and personalised (3P) medicine (see Fig. 3).

## PCa as a multi-factorial disease: general view

**Fig. 4:**
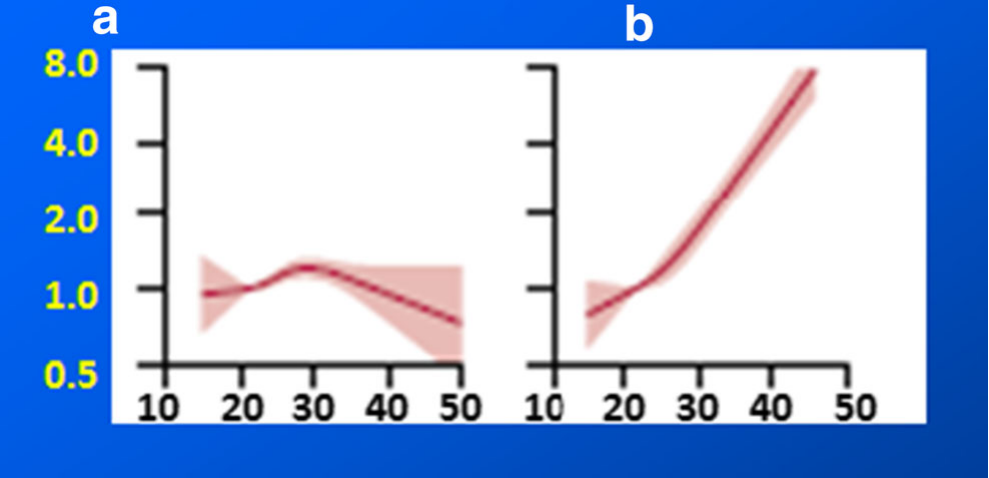
A population-based cohort study of 3.6 million adults performed by Bhaskaran K. with colleagues in the UK demonstrated that the association between BMI and PCa-related mortality (**a**) carries completely different characters compared to many other cancer types, e.g. uterus cancer (**b**); horizontal axis indicates BMI (kg/m^2^) and vertical axis indicates hazard ration (95% CI); the image is adapted from [21]

PCa is a multi-factorial disease resulting from imbalanced interplay between exogenous and endogenous risks against protective factors. The overall picture is extremely comprehensive that perhaps can be illustrated by the complex association between the *Body Mass Index* (BMI) and PCa-related mortality. A population-based cohort study of 3.6 million adults has been recently performed in the UK analysing association of BMI with overall and cause-specific mortality [21]. Noteworthy, whereas uterus, kidney and liver cancers clearly demonstrate the association between overweight/obesity on one hand and exponentially increasing disease-related mortality on the other hand, for the prostate cancer, this association is not that clear (see Fig. 4). Rather in contrast, the demonstrated statistics feature mild decrease in mortality rates for obese PCa patients compared to those with normal and low BMI. However, what is even more important to note in this context, an individual deviation from the average has been demonstrated as particularly pronounced for the BMI-associated PCa mortality indicating a complex interplay between many risks as well as protective factors together contributing to individual patient profiles and creating individual outcomes. Consequently, individualised profiling and patient stratification are crucial for interpretation of the PCa-related data. This indication should be kept in mind as particularly relevant considering all the below listed risks and protective factors related to the disease development and progression.

## Geographic spread of the disease and the mortality-to-incidence ratio

The incidence of PCa is linked to the human development index (HDI). Its age-standardised incidence rate is the highest for high-HDI countries (Australia, Northern and Western Europe, North America), while the age-standardised mortality rate is the highest in low-HDI countries (Middle Africa) [22]. Further, the mortality-to-incidence ratio reflects well the overall quality of PCa care and treatment appropriateness as demonstrated in Table 1.

**Table 1:** The incidence to mortality ratio by country; both crude incidence and mortality rate are expressed as cases or deaths per 100,000 male inhabitants [2]

Country	Crude incidence, cases/100,000 male inhabitants	Crude mortality, deaths/100,000 male inhabitants	Ratio mortality to incidence, %
Ireland	208.80	24.50	11.73
Singapore	117.80	15.40	13.07
USA	131.50	17.70	13.46
South Korea	64.80	8.80	13.58
France	202.50	28.10	13.88
Luxembourg	134.10	18.90	14.09
New Caledonia	120.70	17.70	14.66
Malta	135.00	19.80	14.67
Israel	69.70	10.30	14.78
French Guyana	73.20	11.70	15.98
Italy	151.60	24.50	16.16
Guadeloupe	384.10	63.00	16.40
Czechia	176.50	29.20	16.54
New Zealand	163.70	28.70	17.53
Japan	113.80	20.00	17.57
Puerto Rico	159.70	28.70	17.97
Australia	148.10	26.70	18.03
Spain	139.40	25.50	18.29
Canada	116.80	21.70	18.58
Finland	170.40	33.40	19.60
Brazil	82.00	16.10	19.63
United Arab Emirates	3.70	0.73	19.73
Martinique	329.60	65.10	19.75
Belgium	132.60	26.80	20.21
Brunei	15.20	3.10	20.39
Norway	202.60	43.00	21.22
Switzerland	160.10	34.70	21.67
Hungary	119.50	26.60	22.26
The Netherlands	149.40	33.50	22.42
Austria	130.40	29.40	22.55
United Kingdom	171.60	40.00	23.31
Sweden	211.60	50.40	23.82
Slovenia	170.10	40.60	23.87
Estonia	203.80	50.90	24.98
Cyprus	126.20	31.80	25.20
Germany	154.50	39.10	25.31
Bulgaria	124.40	32.70	26.29
Belarus	76.60	21.60	28.20
Portugal	135.70	38.60	28.45
Greece	117.80	33.80	28.69
Denmark	163.00	47.30	29.02
Latvia	155.70	48.40	31.09
Lithuania	117.20	41.50	35.41
Iceland	104.40	37.20	35.63
Russian Federation	59.90	21.40	35.73
Croatia	116.80	42.70	36.56
Poland	83.70	31.30	37.40
Northern Macedonia	67.70	25.90	38.26
Montenegro	63.40	25.10	39.59
Serbia	74.30	29.60	39.84
Slovakia	89.20	36.60	41.03
Romania	63.60	26.10	41.04
Republic of Moldova	43.00	18.40	42.79
Ukraine	54.60	24.40	44.69
Albania	44.50	20.30	45.62
Bosnia and Herzegovina	54.40	27.10	49.82
Haiti	36.40	23.60	64.84
India	3.70	2.40	64.86
Senegal	12.00	7.90	65.83
Dem. Republic of Congo	13.60	9.00	66.18
South Sudan	11.10	7.40	66.67
Central African Republic	14.10	10.00	70.92
Jamaica	90.80	64.40	70.93
Liberia	15.30	10.90	71.24
Zimbabwe	15.80	11.40	72.15
Lesotho	12.60	9.20	73.02
Guinea	13.80	10.50	76.09

## Non-modifiable risk factors

### Inborn (genetic) cancer predisposition

A family history of PCa is a well-established risk factor of PCa [23]. The findings of the Health Professional Follow-Up Study indicated, based on the follow-ups of 3695 patients, that the probability of the PCa diagnosis increases by 2.3-times in patients with PCa history of both their father and brother. Moreover, if the father or brother were diagnosed with PCa at below or equal to 60 years of age, 2.16- or 1.95-times increased PCa risk has been identified, respectively. An increased risk of EOPCa occurring at age below 65 years was evident in men with a family history of PCa [24]. Five single-nucleotide polymorphisms in combination with a family history of PCa have been reported as having a significant association with PCa incidence. Other familial forms of PCa might be linked to allelic low penetrance mutations identified [25]. Prevalence of the TMPRSS2-ERG fusion gene in PCa is the highest in white American (WA) (49%), followed by Asian (27%) and Afro-American (AA) males (25%) [26]. BRCA2 mutation status in men is an independent risk factor of PCa. BRCA2 mutation carriers are more frequently diagnosed with clinically significant PCa and at younger age compared with non-carriers [27].

### Race and ethnicity

Understanding determinants of ethnic and racial disparities in treatment, mortality, health resource utilisation and associated costs is crucial for developing effective healthcare policies and improving quality of care for PCa patients [28]. Recent studies suggest that ethnicity is an essential risk factor of PCa [29]. In the USA, PCa in AA is biologically and genetically more aggressive compared with PCa in WA [30]; the primary risk is 1.6-times higher, and mortality is more than two-times higher for AA compared with WA males [31]. The secondary risk is higher in AA (18.2%) than WA (13.3%). Mortality rate is generally higher in black populations [32]. Asian males have the lowest incidence of PCa, due to less genetic predisposition as well as more favourable traditional diet and environmental factors as reported [33].

### Accelerated biological ageing

**Fig. 5:**
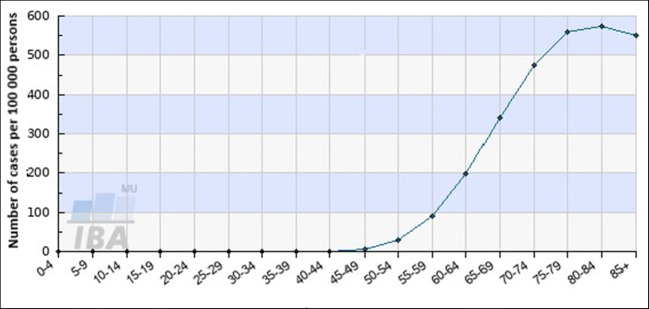
Progressing age is a well-acknowledged non-modifiable risk factor of increasing PCa incidence. In the Czech Republic, this trend is well documented by long-term statistics. Displayed data obtained from the group of 142,994 patients demonstrate age-dependent disease distribution. The data were acquired from the Czech National Cancer Registry (CNCR) managed by the Institute of Health Information and Statistics of Czech Republic (IHIS CR; ÚZIS ČR) and from demographic data database of the Czech Statistical Office (CSO) [34]

Progressing age is a well acknowledged non-modifiable risk factor of increasing PCa incidence at global scale. This trend is well documented by long-term statistics also in the Czech Republic as demonstrated in Fig. 5. The prevalence pick is reached in the age groups 75–85 years; in the age groups 85+ years, the prevalence is decreasing [34].

Consequently, factors contributing to accelerated ageing may further increase PCa risks. Amongst them are both non-modifiable (genetic) factors such as ageing-relevant DNA mutations [35] as well as considered below preventable factors which facilitate ageing, such as smoking and oxidative stress in general [36] causing ageing processes and immunosenescence [37].

### Perturbed immune system

Immunosenescence plays a central role in many tumours including PCa [37]. Chronic inflammation is considered to be indicative for ageing processes, immunosenescence and predisposition to PCa [38]. Further, immunosuppressive PCa subtypes have been demonstrated as particularly challenging to treat [4].

## Modifiable (preventable) risk factors

### Unhealthy lifestyle and inappropriate dietary habits

Unhealthy lifestyle such as smoking, excessive alcohol consumption, sleep deprivation etc., further, in combination with inappropriate dietary habits demonstrate adverse health effects leading to cancer development and progression. In contrast, some specific dietary patterns may have protective function. Below summarised facts provide clear indication for both PCa promoting *versus* inhibiting effects.

### Imbalanced maternal diet and abnormal embryonic development

The intrauterine microenvironment plays a pivotal role in physiologic embryonal development including prostate development, growth and potential predisposition to the PCa later on in life [39]. Using a rat model of maternal exposure to low-protein diet, an impaired prostatic growth associated with prostate carcinogenesis in older offspring has been demonstrated [40]. The authors concluded that gestational low protein and gestational and lactational low protein diets may lead to unbalanced oestrogen-to-testosterone ratio and an increased circulating IGF-1 which altogether increase the prostate carcinoma risks later on in life.

### Toxic environment

It is generally known that a environmental pollution has a negative impact on human health. Chlordecone (CLD) is one of environmental pollutants linked to the PCa development [41]. Another environmental pollutant is the widely discussed Bisphenol A (BPA) also studied in context of PCa. Corresponding dosage effects have been demonstrated: the higher exposure to BPA is linked to higher PCa risks. To this end, the overall BPA exposure is clearly modifiable, e.g. by drinking water kept in plastic containers or eating meat and fish products in coated food cans [42]. Animal studies showed that the prostate is particularly sensitive to BPA-induced transcriptomic reprogramming, immune disruption and aberrant growth dysregulation [43].

Further environmental pollutants impacting the prostate health are pesticides. An Agricultural Health Study (AHS) in the USA revealed strong association between the utilisation of insecticides and/or herbicides and aggressive type of PCa incidence (GS ≥ 7), when the period of exposure was longer than 4 years [44].

Further, heavy metals have been demonstrated to increase PCa risks. Detailed investigations have been performed for cadmium (Cd) which is considered as one of the main pollutants in economically developed countries contributing to the development of many types of cancer, including PCa [45]. Considering other heavy metals, increased serum levels of As, Zn, Mn and Sb were strongly associated with PCa risks [46].

Finally, specific professional occupation linked to the application of toxic chemicals as well as abnormal physical and emotional strain, such as in the case of firefighting professionals, both have been demonstrated to predispose the exposed individuals most frequently to the gastric carcinoma and PCa [47].

### Imbalanced stress conditions: the multi-factorial role of ROS and RNS

Excessive release of reactive oxygen species (ROS) and reactive nitrogen species (RNS) have been identified as initiators, mediators and regulators of cellular oxidative stress (OxiS). OxiS can damage almost all biomolecules including chromosomal and mitochondrial DNA [48]. Recent studies suggest that oxidative damage plays an important role in PCa pathology. Several parameters of oxidative damage and antioxidant defence were compared in the group of high-risk patients diagnosed having precursor high-grade intraepithelial neoplasia lesions and in the group of age-matched healthy controls. The concentration of the product of the oxidative damage 8-hydroxydeoxyguanosine (8-OHdG), an oxidised nucleoside of DNA, was significantly higher in high-risk subjects, whereas antioxidant defence substances—glutathione-S-transferase (GST) and glutathione (GSH)—were detected at significantly higher concentration in healthy controls [49]. Specifically, oxidative stress damage can promote castration-resistant PCa via the androgen receptor (AR)-dependent pathway [50].

Nitric oxide (NO) is a signalling molecule that plays an important role in both - physiologic processes and cancer promotion. Regarding the latter, it has been suggested that low levels of NO are cancer promoting, while high levels of NO are protective against cancer. Both ROS and RNS can be carcinogenic by modifying the inflammatory status as well as influencing cellular lipid structures, angiogenesis and anti-apoptotic pathways, amongst others. For example, low concentrations of NO can result in the redox imbalance, increased inflammation and damage to sub-cellular components accelerating neoplastic process. NO production modifies the sensitivity of AR; in turn this receptor becomes sensitised to lower levels of androgens in the microenvironment of the prostate. Those effects are currently considered for targeted therapies in PCa management [51].

### Smoking

Adverse health effects of smoking should be considered in the context of both metabolic changes and detoxification processes [52]. Consequently, functional polymorphisms in genes involved in metabolism and detoxification at the individual level significantly modify PCa risks in smokers [53]. Elevated levels of circulating androsterone and testosterone in male smokers might increase PCa risk and contribute to cancer progression [54]. A meta-analysis of 24 studies suggested that the level of cigarette exposure is directly linked to mortality by 24–30% increased risk of death from PCa in heavy smokers compared with non-smokers. Both former and persistent smokers had an increased risk of PCa development [55]. Further, smoking influences recurrence rates which are 5.2-times higher in smokers compared with lifelong non-smokers, and in former smokers, the recurrence rate is 2.9-times higher [56]. The comprehensive study analysed during 22 years 5366 PCa patients: smoking prior to diagnosis was associated with 61% increased risk of PCa mortality. This study also found that men, who reported having quit smoking more than 10 years ago, demonstrated PCa mortality risks similar to those who had never smoked [57].

### Sleep disorders and night shift work

Patients with sleep disorders (SDs) have increased primary PCa risk: the adjusted hazard ratio corresponds to 1.42-times higher risk and specifically in the group aged ≥ 65 years to 1.35-times higher risk [58]. Further, SDs are common as a post-treatment complication [59]. Finally, night shift work has been demonstrated to be positively associated with the PCa incidence [60].

### Sexually transmitted diseases

Sexual activity is a factor which may play a role in the risk of prostate cancer; however, the exact mechanism is unknown [61]. Further, sexually transmitted diseases (STDs) are suspected of playing a role in PCa risk. A recent meta-analysis provides evidence of higher PCa incidence in men with a history of gonorrhoea and HPV, amongst other STDs [62, 63]. Men diagnosed with trichomoniasis infection were 3-times more likely to die of PCa indicating a link between the detected infection and PCa aggressiveness [64].

### Hormonal dysregulation

Hormonal regulatory axis affects nuclear receptors and transcriptional regulators, thereby changing the gene expression important for tumour development, progression and metastasising [65]. Androgen receptor (AR) signal is known as a powerful driver of PCa progression. A study focused on the relation between TT serum levels and PCa prognosis revealed the serum level of TT 2–8 ng/mL to be the optimal one, whereas patients with too low (< 2 ng/mL) or too high (≥ 8 ng/mL) TT serum levels demonstrated castration-resistant PCa progressing to advanced stages more frequently and with poorer prognosis [66].

Insulin-like growth factor 1 (IGF-1) plays a crucial role in PCa pathophysiology [67]. An elevated IGF-1 serum levels could be detected 5 years before the PCa diagnosis [68]. Increased levels of cortisol, estradiol and leptin are characteristic for PCa patients [69]. The leptin release is regulated by other hormones (insulin, glucocorticoids, estradiol, testosterone, somatostatin, IGF-1). Leptin subsequently stimulates the synthesis of oestrogens. Further, adverse effects of hormone therapy have to be taken into account: PCa patients undergoing androgen deprivation therapy (ADT) are at increased risk of diabetes, metabolic syndrome and cardiovascular diseases [70].

### Vasectomy

Vasectomy is an intervention commonly practised in the USA and Europe. Although some studies have associated vasectomy with potential PCa risks [71], clear pathomechanisms for such a link have not yet been described [72].

### Chronic inflammation

Urinary tract infections (UTI) such as cystitis and urethritis are associated with a primary PCa risk. The primary risk of PCa increases with recurrent low urinary tract infection (LUTI) linked to chronic inflammation [73, 74].

Understanding the relationship between the urinary microbiome set-up and prostatic chronic inflammation is crucial in developing PCa preventive strategies. Chronic inflammation initiated by microbial species persisting in the urinary tract promotes prostate inflammatory atrophy which may result in the PCa development [75]. In current diagnostic practice, prostate tissue biopsy is used to confirm PCa diagnosis. However, long-term observations suggest that inflammation by drawing tissue biopsy can predict the PCa development. Consequently, non-invasive analytical approaches such as the liquid biopsy analysis are currently under development avoiding adverse health effects linked to drawing tissue biopsy [76, 77].

Further, low-grade inflammation could be associated with the presence of more aggressive forms of PCa [78]. Non-bacterial prostatitis was induced in an animal model after 5 days of oestradiol administration accompanied with significantly increased IL2 and PCA3 mRNA expression levels. To this end, utilising anti-inflammatory effects of the orange peel extract, containing selenium, the inflammatory process has been suppressed accompanied by decreased levels of the above parameters measured [79]. The Prostate Cancer Study throughout Life (PROCA-life) investigating potential relationship between high sensitive CRP (hs-CRP) levels and white blood cell count (WBC) demonstrated that a significant increase in hs-CPR levels was associated with high PCa risks. An increased systemic inflammatory score (WBC, hs-CRP) was associated with a pronounced predisposition to metastatic PCa [80].

### Collateral pathologies

#### Metabolic syndrome

Metabolic syndrome (MetS) is associated with increased primary PCa risk but not with GS value. Higher circulating CRP levels were positively correlated with higher GS and secondary PCa risk [81]. Further, MetS history is associated with poor prognosis of PCa outcomes, particularly in the case of aggressive PCa subtypes [82]. Independently of overweight, hypertension and type 2 diabetes both are associated with higher risk of aggressive PCa [83, 84].

#### Chronic obstructive pulmonary disease

Patients with chronic obstructive pulmonary disease (COPD) have 1.62-times increased primary risk of PCa development compared with non-COPD patients. Both the systemic hypoxic condition and systemic inflammation associated with COPD have been proposed as promoting PCa progression and secondary risks. Treatments by short-acting muscarinic antagonists (SAMAs) and short-acting beta-agonists (SABAs) increase the risk of primary PCa. In contrast, primary risk of PCa decreases, when statins are implemented to COPD patients [85]. Increased levels of tPSA and fPSA in blood serum accompany COPD progression, due to systemic hypoxic effects; contextually PSA levels in COPD patients should be adequately interpreted [86]. To this end, phenotypes linked to systemic ischemic-hypoxic effects may predispose affected individuals to PCa development and particularly aggressive disease progression. This consideration should be taken into account, for example, in the case of individuals with Flammer syndrome phenotype demonstrating systemic ischemic-hypoxic effects; phenotyping should be further linked to the disease-specific molecular patterns for the disease prediction and prognosis [18, 87].

### Abnormal BMI

Abnormal BMI affects overall risks and mortality rates in a large spectrum of pathologies including several cancer types [88]. Information available is controversial regarding BMI which might be optimal against PCa development and related mortality that leads to a conclusion that BMI is a highly individual parameter in relation to PCa.

Doubtless obesity negatively impacts microcirculation and sex steroid hormones, therefore, influencing the aggressiveness of PCa [89]; data from several national SA surveys reported a positive association between obesity on one hand and PCa incidence and progression on the other hand [90]. The insulin resistance typically found in obese patients leads to chronically elevated blood levels of insulin and IGF-1 that contributes to cancer development and progression. Generally, malignancies are more difficult to detect in obese men [91]. Further, an increased prostate volume makes it more difficult to detect cancer by drawing tissue biopsy. Therefore, early stages of PCa are less likely to be diagnosed in obese men [92]. Finally, obesity should be considered in the context of individual patient profiling including other PCa risks such as family history, age, race etc. [93].

On the other hand, a study focused on PCa-specific mortality and overall mortality in relation to BMI demonstrated statistically significant risk of PCa-specific mortality and overall mortality in the group with high BMI (≥27.5 kg/m^2^) as well as in the group with low BMI (<22.5 kg/m^2^) compared with the reference group (BMI 22.5–25 kg/m^2^) [94]. However, data collected from 22 clinical trials showed that BMI ≥ 25 kg/m^2^ was associated with better overall survival amongst PCa patients compared to those with BMI < 25 kg/m^2^ [95]. Obviously individualised patient profiling and precise patient stratification is essential for PCa-related data interpretation as stated above (see “PCa as a multi-factorial disease: general view”).

### Abnormal alcohol consumption

Extensive alcohol consumption may increase risks of PCa development [96]. Researchers suggested that risk ration (RR) of PCa elevates with increasing amount of alcohol drinks per day [97]. The effect of alcohol is modulated by polymorphisms in genes encoding for enzymes responsible for ethanol (alcohol dehydrogenases) and folate metabolism as well as for the DNA repair [98]. On the other hand, moderate alcohol consumption has been demonstrated as slightly decreasing PCa-related mortality compared with abstainers. Further, PCa-diseased patients show lower risk of disease progression by moderate consumption of red wine in some populations studed [99].

### Saturated animal fat

Animal fat consumption is positively associated with PCa incidence and mortality [100]. High-energy intake, lipid metabolism and an increase in testosterone altogether could explain possible biological mechanisms by which saturated animal fat impacts PCa incidence [101]. Likewise, calcium intake has been shown to influence PCa cell growth and susceptibility to apoptosis. Small imbalances in calcium homeostasis could, therefore, result in increased proliferation, differentiation and apoptosis of PCa cells [102].

### Eggs (choline)

There is a controversial presentation of potential association between eggs consumption and PCa risks in the literature. On the one hand, several studies have associated high choline intake with an increased risk of the disease development [103], aggressiveness [104] and/or recurrence of PCa [105]. Another meta-analysis recorded no PCa risks observed [97]. Obviously individualised patient profiling and precise patient stratification is essential for PCa-related data interpretation as stated above (see “PCa as a multi-factorial disease: general view”).

### Processed red meat

The World Health Organisation (WHO) classified processed meat as “carcinogenic to humans” and red meat as “potentially carcinogenic to humans” referring to the evidence-based association between an extensive red meat consumption and increased risk of advanced PCa [106]. While several studies have reported a positive association between processed red meat consumption and risk of advanced and/or fatal PCa [107], others concluded no direct association [108]. Obviously individualised patient profiling and precise patient stratification are essential for PCa-related data interpretation as stated above (see “PCa as a multi-factorial disease: general view”).

## PCa-relevant prevention

Inborn genetic predisposition is characteristic for minor portion of cancer cases. In contrast, modifiable risk factors contribute to the development of the majority of cancers. Contextually, general and targeted prevention is in focus of the cost-effective approach by 3P medicine.

### Regular physical activity and well-controlled stress exposure

Vigorous physical activity is defined as activity that causes sweating, increased heart and respiratory rates. Typically, vigorous activities like jogging, biking, swimming, and bicycling are considered [109]. A comprehensive Health Professionals Follow-Up Study (HPFS) has demonstrated that men performing vigorous activity 3 or more hours weekly are at lower risk (61%) to die from PCa compared with men performing vigorous activity less than 1 h per week [110]. CaPSURE study led to similar conclusions: men who walked 3 or more hours per week at a brisk pace (≥ 3 mph) are at 57% lower risk of PCa recurrence compared with men who walked less than 3 hours per week at an easy pace (< 2 mph) [111]. A Swedish study with 4623 men included stated that men who walked or biked ≥ 20 min/day are at 36% lower risk of PCa-related mortality compared with men who walked or biked < 20 min/day [112]. The protection mechanisms by vigorous activity are complex including improved microcirculation, anti-stress and ant-inflammatory effects. To this end, antioxidants are essential to suppress tumorigenesis at molecular and cellular levels [113, 114]. Contextually, dietary supplements with genoprotective properties are strongly recommended for cancer prevention [114].

### Personalised nutrition

#### Fish

High fish intake, especially containing high levels of omega-3 fatty acids, is associated with decreased incidence [115], progression [116] and recurrence of PCa [117]. To this end, one of the studies in the area proposed that individuals consuming fish-rich diet are well informed about healthy lifestyle taking advantages also from diagnostics such as regular PSA testing [118]. Further studies demonstrated that fish and its components possess PCa protective properties against any stage of tumour development and progression: men consumed fish oil 4–6 weeks prior to the planned PCa surgery demonstrated inhibited prostate tumour growth [119]; lower rates of PCa recurrence have been demonstrated for PCa diagnosed men consuming fish-rich diet [120].

#### Tomatoes and lycopene

Epidemiological studies report plant-based foods including cruciferous vegetables, tomatoes, garlic, pomegranate and green tea as associated with a significant reduction in the progression of PCa [121, 122]. Regular intake of cooked tomatoes is associated with a decreased primary PCa risk, whereas no such association is observed for raw tomatoes intake [123]. Tomatoes are rich on lycopene contents. Lycopene and other carotenoids have a number of anti-cancerous biological effects including anti-oxidative, geno-protective, anti-proliferative and anti-angiogenic properties, and inhibition of cancer cell growth [124–126].

#### Garlic

Traditional garlic consumption is highly protective against the primary PCa risk. Accordingly to currently provided recommendations, the minimal daily dose of garlic intake is 10 g [127].

#### Dark-skinned grapes (stilbenes)

Grape powder extract (GPE) contains resveratrol or its natural analogue—pterostilbene. Resveratrol and pterostilbene are stilbenes involved in numerous biochemical and molecular pathways preventing PCa progression and metastatic disease [128]. In vitro studies showed that GPE concentrations higher than 100 μg/mL inhibit viability and growth of PCa cells [129].

#### Pomegranate peel extract, black berries, wild strawberries and raspberries (ellagic acid, EA)

Pomegranate peel extract, black berries, wild strawberries and raspberries are rich on EA which is effectively protecting against PCa [130]. EA shows anti-proliferative and pro-apoptotic activity and suppresses tumour cell migration, extracellular matrix invasion as well as angiogenesis—all are crucial for tumour progression and metastatic disease [131].

#### Broccoli and other cruciferous vegetables (sulforaphane, SFN)

Broccoli and its active compound SFN demonstrate highly protective properties useful for targeted prevention against primary PCa risks and possibly for secondary risks mitigation [132]. Other commonly consumed cruciferous vegetables include cauliflower, cabbage, brussels sprouts, kale, mustard greens and chard greens. In vitro experiments and animal studies suggest that metabolites of cruciferous vegetables, isothiocyanates and indoles may detoxify carcinogenic compounds, suppress cancer cell proliferation and promote apoptosis of cancer cells [133]. Consequently, diets rich on cruciferous vegetables are associated with decreased risks to disease on aggressive PCa subtypes [120].

#### Red onions, white onions, green hot peppers, elderberries and cranberries (quercetin, QCT)

Red onions, white onions, green hot peppers, elderberries cranberries are rich on QCT contents. Similarly to SFN, QCT demonstrates highly protective properties useful for targeted prevention against primary PCa risks and possibly for secondary risks mitigation [134]. QCT is effective in suppressing proliferation and inducing apoptosis in androgen-independent cell line [135]. However, QCT bioavailability is limited, as it is poorly water soluble [136]. Consequently, QCT-loaded nanomicelles are more effective for prevention than free QCT.

#### Green tea (EGCG)

The incidence of PCa recorded in East-Asian countries is significantly lower compared with Western countries that has been associated with a traditionally abundant consumption of green tea there. Green tea is rich on catechin (epigallocatechin-3-gallate, EGCG) demonstrating chemopreventive effects against PCa development and metastatic disease in experimental models [137–139].

#### Coffee

A series of studies reported a strong association between regular coffee consumption and significantly reduced risks of the PCa development, progression, recurrence and related mortality [140–142]. Noteworthy, results were comparable for caffeinated and decaffeinated coffee. Several biological mechanisms have been proposed for anti-PCa effects linked mainly to strong antioxidant properties of coffee.

#### Curcumin

Curcumin slows down proliferation and induces apoptosis in the PCa cells. Further, curcumin demonstrates strong anti-angiogenic properties and downregulates AR expression. Its regular consumption as a dietary supplement is highly recommended [127].

### Gut microbiota in PCa pathogenesis and outcomes: application of pre- and probiotics

Gut microbiota have been demonstrated as a strong contributor to tumorigenesis and may, further, influence the tumour environment [143]. Specifically for PCa, an increase in the abundance of specific gut bacteria has been reported [144]. On the other hand, diet composition and lifestyle have a direct and profound effect on the gut bacteria. These reciprocal effects have been clearly demonstrated for disease predisposition, treatment efficacy and outcomes utilising animal models [145]. Contextually, clinical implementation of targeted pre-biotics and individualised profile adapted probiotics have been proposed [145]. Probiotic therapy in overall PCa management is currently under consideration [146].

### Vitamins and trace elements

Vitamins A, C, D, E, K and folate have been demonstrated as potentially affecting PCa pathogenesis and progression [144, 147–152]. However, there is a consensus which can be recognised in all publications that protective effects are highly individual and should be considered in context of other risks utilising questionnaires, individualised patient profiling and multilevel diagnostic approach. In detail, high folate levels may protect against PCa but low folate levels may increase risk of metastatic PCa [147]. Vitamin C has been identified as a promising anti-cancer agent proposed for PCa treatment, due to its scavenging activity against excess of ROS and under oxidative stress conditions [148]. Vitamin D demonstrates anti-cancer properties in general [149, 150] being also protective specifically against PCa development and progression [151–153]. Vitamin E demonstrates anti-PCa effects [154]. Vitamin E consists of tocopherols and tocotrienols; α-tocopherols supplementation was found to diminish PCa risks [155, 156]. Vitamin K inhibits prostate cancer cells, and altered expression rates of vitamin K-dependent proteins in prostate tumours have been linked to their aggressiveness and progression [157]. Trace elements play a multi-faceted role in central biologic processes. PCa relevant aspects include anti-inflammatory, anti-proliferative and pro-apoptotic effects, amongst others. A series of studies demonstrated that PCa risks can be significantly reduced by consumption of a selenium-rich diet [158]; however, selenium supplementation of 140 or more μg/day after diagnosis of nonmetastatic prostate cancer may increase risk of prostate cancer mortality [159]. Recently published data suggest selenite as an effective compound for the therapy of apoptosis-resistant prostate cancer, due to its anticancer effect by inducing apoptosis in androgen-dependent LNCaP prostate cancer cells [160].

## Concluding remarks and 3PM related outlook

**Fig. 6:**
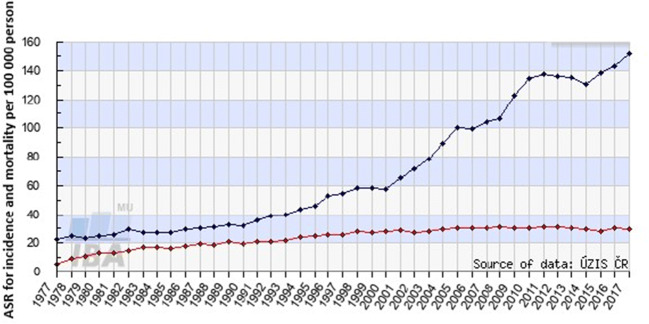
PCa incidence (blue line) and related mortality (red line) in Czech Republic during 50 years (1977–2017); ASR (age-standardised rate) per 100,000 person; displayed data were obtained from the group of 142,994 patients; the data were acquired from the Czech National Cancer Registry (CNCR, managed by the Institute of Health Information and Statistics in Czech Republic (IHIS CR; ÚZIS ČR) and from demographic data database by the Czech Statistical Office (CSO) [161]

**Fig. 7:**
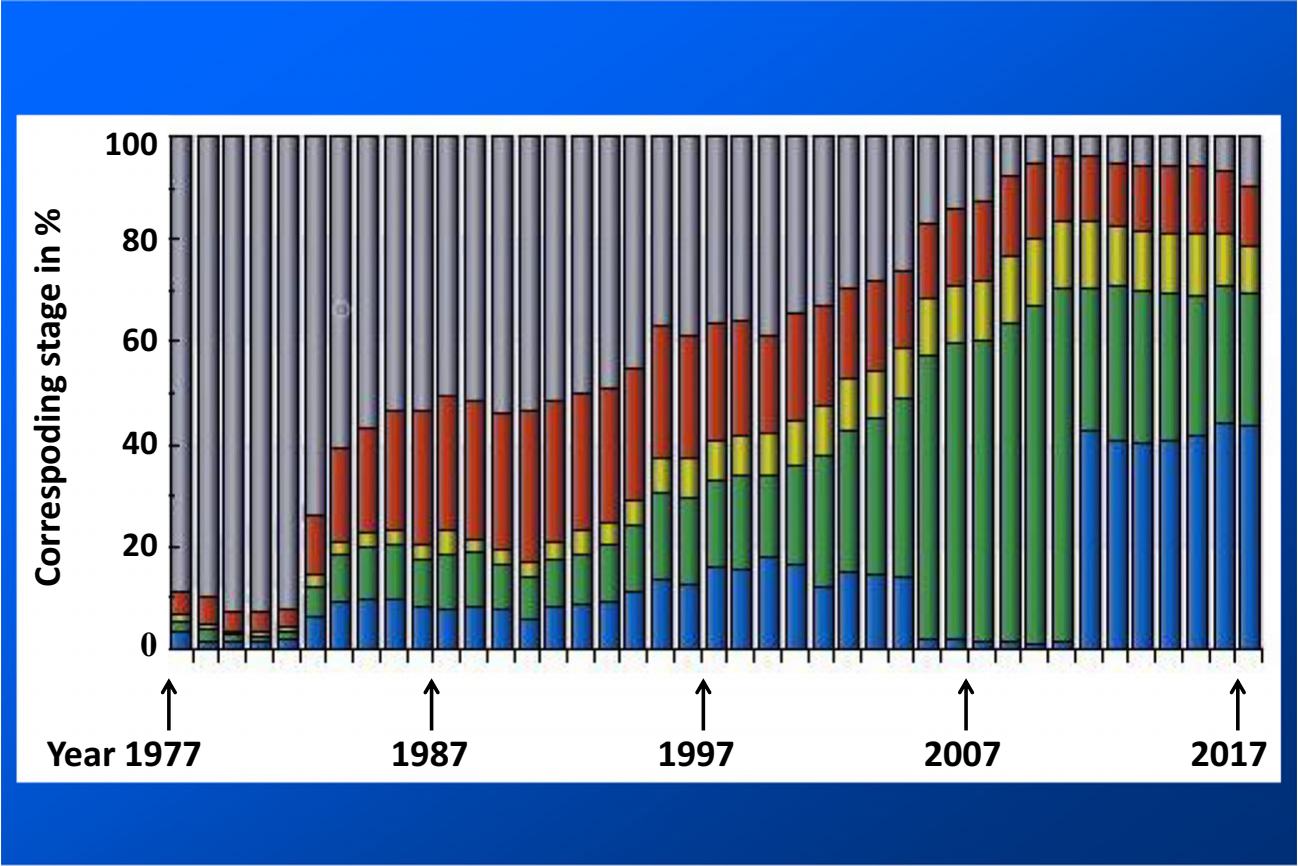
PCa clinical stage stratification in Czech Republic evolving during 50 years of monitoring (1977–2017); stage I (blue), stage II (green), stage III (yellow), stage IV (red), unknown stage (grey); displayed data obtained from the group of 142,994 patients; the data were acquired from the Czech National Cancer Registry (CNCR, managed by the Institute of Health Information and Statistics in Czech Republic (IHIS CR; ÚZIS ČR) and from demographic data database by the Czech Statistical Office (CSO) [162]

In the early twenty-first century, societies around the world are facing the paradoxal epidemic development of PCa as a non-communicable disease: PCa is the most frequently diagnosed cancer for men in several countries such as the USA [4]. Permanently improving diagnostics and treatments in the PCa management causes an impressive divergence between, on one hand, permanently increasing numbers of diagnosed PCa cases and, on the other hand, stable or even slightly decreasing mortality rates as demonstrated in Fig. 6, exemplifying the 50-year-old evolution in Czech Republic. As further detailed in Fig. 7, the input by uncharacterised and stage IV tumours has been minimised particularly during the last decade that consequently improved outcomes [161, 162].

PCa patients are benefiting a lot from personalisation of medical services: the general approach by the radical castration has been revised for several subtypes of PCa, since keeping urinary and sexual functions intact allows for significantly higher quality of life for many PCa patients without diminishing the survival rates [163].

Three pillars are currently involved in the standard PCa diagnostics: prostate biomarker panel, medical imaging and tissue biopsy [164]. Current biochemical panel is based on the measurement of the total prostate-specific antigen (tPSA). To this end, PSA derivatives are frequently used as individual biomarkers and/or as the part of combined parameters: free PSA (fPSA), PSA precursor [-2]proPSA, percentage of fPSA (%freePSA = (fPSA/tPSA)*100), Prostate Health Index (PHI) (PHI = ([-2]proPSA/fPSA) x √tPSA) and 4Kscore which combines fPSA, intact PSA, tPSA and kallikrein-related peptide 2 (hK2). Prostate cancer antigen 3 (PCA3, prostate-specific non-coding mRNA) is involved in the biomarker set. The detection is performed in urine based on the prostate massage approach releasing biomarkers to urine [165, 166].

Comprehensive medical imaging is utilised in the overall PCa management: transrectal ultrasound (TRUS), multi-parametric magnetic resonance imaging (mpMRI) to evaluate local staging and hybrid imaging methods: ^18^F-methylcholine and ^68^Ga-PSMA-11 positron emission tomography (PET)/MRI [167, 168].

Main indication for initiating PCa treatment is the histological analysis of the biopsy. The TRUS-navigated biopsy is used as a basic procedure. The second variant is the cognitive biopsy in which the result of the imaging technique, most often mpMRI, is known. Currently, the preferred procedure is the fusion biopsy, where mpMRI and TRUS images are merged by software. MRI information is used to guide biopsy cores to suspicious areas within the prostate. PET/TRUS and PET/MRI/TRUS fusions have also been tested [169]. Based on biopsy results, the Gleason score (GS) was used since the 1960s as the main grading system for PCa cell assessment GS got revised in 2016 by the International Society of Urological Pathology (ISUP) for using a new grading system and scaling (5 ISUP Grades) [170].

Still, several aspects are waiting for innovate solutions, which obviously can be provided only if the paradigm shift from reactive to predictive, preventive and personalised medicine will get implemented to the overall PCa management as detailed below.

A. PCa belongs to the cancer types with highest incidence

The PCa incidence is annually increasing. Corresponding economic burden is enormous [171]. Being the most common malignancy affecting male subpopulation in  the USA, PCa creates enormous financial burden on the USA healthcare system [172]. The NCI estimated the costs associated with PCa diagnosis and treatment as high as $11.85 billion in 2010 being the fifth most costly cancer. Moreover, the costs of treating PCa are currently increasing more quickly than those of any other cancer [173]. Several research groups conclude that imprecise selection of patients for treatment increases the overall number of overtreated PCa cases and corresponding unnecessary costs [174–176]. To this end, about $1.32 billion per year could be saved in the USA by not treating approximately 80% of low-risk PCa cases who would never die of the disease [176]. Undertreatment may be an issue as well in the case of patients for whom the disease recurrence has been underestimated [175]. Implementing individualised patient profiles and adapted treatment algorithms would make currently too heterogeneous landscape of PCa treatment costs more transparent providing clear “roadmap“ for the cost-saving.

B. PCa is a systemic multi-factorial disease

PCa is a systemic multi-factorial disease. Consequently, predictive diagnostics by liquid biopsy analysis is instrumental for the disease prediction, prevention and curative treatments at early stages. Further, the absolute majority of PCa patients suffer from comorbid conditions as demonstrated for oncologic patients in general [177]. To this end, both the disease modelling [178] and patient stratification should carefully consider this aspect in terms of adequate prediction and prognosis as well as targeted prevention and personalised treatment algorithms. Obviously metastatic castration-resistant PCa cases need detailed phenotyping and molecular analysis to elaborate on the subtype-specific diagnostic and treatment targets. Further, due to systemic effects linked to the pathology, liquid biopsy might be particularly useful for early and predictive PCa diagnostics [87].

C. The incidence of metastasising PCa is rapidly increasing

Exemplified by trends observed in the USA, metastatic PCa began to increase by 0.58% per year after 2008 and accelerated to 2.74% annually following the 2012. The pattern was magnified amongst men aged below 69 years. Prognosis in 2025 is that men aged 45–54 years might demonstrate more rapid annual increase by 2.29% versus men aged 55–69 years by 1.53% per year reaching the annual burden increase of 42% by 2025 [179]. To this end, one of the evident deficits is the reactive character of medical services provided to populations [18]. The paradigm shift from reactive to predictive, preventive and personalised (3P) medicine would provide adequate solution—the concepts presented by the European Association for Predictive, Preventive and Personalised Medicine [180]. Innovative screening programmes might be useful to identify persons in suboptimal health conditions before the clinical onset of metastasising PCa. Strong predisposition to systemic hypoxic conditions and ischemic lesions (e.g. characteristic for individuals with Flammer syndrome phenotype) [18] and low-grade inflammation might be indicative for specific phenotyping and genotyping [87, 181, 182]. Liquid biopsy tests for CTC enumeration and their molecular characterisation are considered to be useful for secondary prevention of metastatic disease in PCa patient cohorts. To this end, Mandel P.C. with colleagues [183] demonstrated that the prognostic value of CTCs was highest using Harrell’s C compared with routinely used biomarkers (prostate-specific antigen, lactate dehydrogenase and bone-specific alkaline phosphatase), while the highest C-index was achieved by combining conventional markers with CTC enumeration. After progression to metastatic castration-resistant PCa, CTC enumeration was prognostic for overall survival [183]. The authors conclude that in their study, the CTCs’ number in liquid biopsy was highly prognostic at any step of the disease-related analysis being more powerful indicator than other commonly used biomarkers.

D. Rapidly increasing incidence rates are characteristic for adolescents and young adults aged 15–40 years [1, 14]

Multi-factorial risks for these trends have been proposed. Consequently, multi-level diagnostics including phenotyping and multi-omics is considered to be the most appropriate tool for risk assessment, prediction, targeted prevention and prognosis [184]. To this end, patients with early onset prostate cancer pose unique challenges. Current data suggest that early onset prostate cancer is a distinct phenotype from both an aetiological and clinical perspectives that deserves further attention.

## Abbreviations

[-2]proPSA: Subunit of precursor of the prostate-specific antigen
8-OHdG: 8-hydroxydeoxyguanosine
AA: Afro-American men
AHS: Agricultural health study
ACS: American Cancer Society
ADT: Androgen deprivation therapy
As: Arsen
AR: Androgen receptor
ASR: Age-standardised rate
BMI: Body Mass Index
BPA: Bisphenol A
BPH: Benign prostate hyperplasia
BRCA: Breast cancer gene
CaPSURE: Cancer of the Prostate Strategic Urologic Research Endeavor study
CI: Confidence interval
Cd: Cadmium
CLD: Chlordecone
CNRC: Czech National Cancer Registry
Co: Cobalt
COPD: Chronic obstructive pulmonary disease
CSO: Czech Statistical Office
Cu: Copper
DRE: Digital rectal examination
EA: Ellagic acid
EGCG: Epigallocatechin-3-gallate
EOPCa: Early onset prostate cancer
EPMA: European Association for Predictive, Preventive and Personalised Medicine
EU: European Union
fPSA: Free fraction of the prostate-specific antigen
GSH: Glutathione
GST: Glutathione S-transferase
GPE: Grape powder extract
GS: Gleason score
hs-CRP: High sensitive CRP
HDI: Human Development Index
hK2: Kallikrein-related peptide 2
HPFS: Health Professionals Follow-Up Study
IARC: International Agency for Research on Cancer
IGF: Insulin-like growth factor
IGFBP: Insulin-like growth factor-binding protein
IHIS CR: Institute of Health Information and Statistics of the Czech Republic
IL2: Interleukin 2
ISUP: International Society of Urological Pathology
LNCaP: Cell line of androgen-sensitive human prostateadenocarcinoma cells
LOPCa: Late-onset prostate cancer
LUTI: Lower urinary tract infection
MetS: Metabolic syndrome
Mn: Manganese
mpMRI: Multi-parametric magnetic resonance imaging
mtSNVs: Mitochondrial single-nucleotide variant
NCI: National Cancer Institute
Ni: Nickel
NO: Nitric oxide
OR: Odds ratio
OS: Overall survival
OxiS: Oxidative stress
Pb: Lead
PCa: Prostate cancer
PCA3: Prostate cancer antigen 3
PET/MRI: Positron emission tomography/magnetic resonance imaging
PHI: Prostate Health Index
PROCA-life: Prostate Cancer Study throughout Life
PSA: Prostate-specific antigen
QCT: Quercetin
RNS: Reactive nitrogen species
ROS: Reactive oxygen species
ROCK1: Rho-associated coiled-coil containing kinase 1
RR: Relative risk
SA: Sexual activity
SABAs: Short-acting beta-agonists
SAMAs: Short-acting muscarinic antagonists
Sb: Tin
SD: Sleep disorder
Se: Selenium
SFN: Sulforaphane
STDs: Sexually transmitted diseases
TNM classification: Tumour (T), Nodes (N), Metastases (M) classification system
tPSA: Total prostate-specific antigen
TRAMP: Transgenic adenocarcinoma of the mouse prostate
TRUS: Transrectal ultrasound
TT: Total testosterone
USA: The United States of America
UTI: Urinary tract infection
ÚZIS ČR: Institute of Health Information and Statistics of the Czech Republic
VDR: Vitamin D receptor
WA: White American men
WBC: White blood cell count
WCRF: World Research Cancer and Fund
WHO: World Health Organisation
Zn: Zinc
